# Editorial: Stimuli-Responsive Emissive Organic and Metal-Organic Compounds

**DOI:** 10.3389/fchem.2022.946617

**Published:** 2022-06-06

**Authors:** Zhao Chen, Dian-Dian Deng, Riina Aav, Victor Borovkov, Yue Sun

**Affiliations:** ^1^ Jiangxi Key Laboratory of Organic Chemistry, Jiangxi Science and Technology Normal University, Nanchang, China; ^2^ Department of Chemistry and Biotechnology, Tallinn University of Technology, Tallinn, Estonia; ^3^ State Key Laboratory of Separation Membrane and Membrane Process, School of Chemistry, Tiangong University, Tianjin, China

**Keywords:** functionalized materials, stimuli-responsive, luminogenic materials, aggregation-induced emission, fluorescent organic compounds, luminogenic metal complexes, chemosensors

Light is an indispensable part in the production and living of human society, and the development of light-emitting materials is of great significance for high-tech innovations. Indeed, the exploitation of high-performance luminogenic materials has opened a new avenue to scientific advancement and societal development. For example, Chen et al. reported a fluorene-based dinuclear gold(I) complex. This complex demonstrated a remarkable aggregation-induced white-light emission feature, and it emitted high brightness solid-state and thin-film white luminescence. Furthermore, its thin-film white-light emission quantum yield was up to 65.42%, which enabled this gold(I) complex to serve as a potential candidate for white OLED (Chen et al., 2014). Benzobisthiadiazole (BBT) belongs to a typical near-infrared emissive unit, and the photoluminescence (PL) of numerous BBT derivatives lie in the second near-infrared wavelength ranges (NIR-II, 1,000–1,700 nm). The NIR-II-emissive nature of this type of compounds is beneficial to biomedical applications. Sun et al. elaborately designed a multipurpose nano-agent by incorporating a supramolecular Pt (II) metallacycle and a BBT-modified NIR-II-emissive organic dye into multifunctional melanin dots possessing photoacoustic and photothermal properties, and this prepared nano-agent displayed superior dual-modal imaging-guided chemo-photothermal synergistic therapy effect (Sun et al., 2019). Ding et al. prepared a high-efficiency nanotheranostic agent by introducing a hexagonal organoplatinum (II) metallacycle and a BBT-based NIR-II molecular dye into a FDA-approved commercial theranostic agent, and this obtained nano-cocktail could be applied for effective cancer imaging and therapy (Ding et al., 2019). Huang et al. summarized a variety of benzobisthiadiazole-functionalized semiconducting polymers, and their resulting nanoparticles could be used for NIR-II photoacoustic imaging. Luminescent chemosensors possess a number of advantages in practical applications, such as high sensitivity and selectivity, versatility, and superior real-time detection capacity. In order to prepare multifunctional luminescent chemosensors, plenty of specific functional recognition units have been introduced to luminogenic organic compounds and organometallic complexes, and the resultant functionalized molecules are capable of forming PL responses to metal ions, pH, anions and biomolecules. For example, Hu et al. synthesized a pyrimidinone-containing Schiff base FPS by a classical aza-Wittig reaction, which could be applied to selectively and sensitively detect Zn^2+^ with the limit of detection of 1.19 × 10^–8^ mol/L. Furthermore, the resulting FPS-Zn^2+^ could further serve as a high-efficiency chemosensor for Cu^2+^. Therefore, this interesting Schiff base molecule showed sequential responses towards Zn^2+^ and then Cu^2+^. Han et al. prepared Pb^2+^ responsive water-soluble orange light-emitting Cu-In-Zn-S quantum dots stabilized by a glutathione via a simple hydrothermal way. This obtained quantum dots are expected to be effectively used for bioimaging and biolabeling because of the properties of low cytotoxicity and good biocompatibility, and the responsive mechanism of Pb^2+^ is attributed to the formed hydrogen bonding or van der Waals forces.

In the area of luminogenic materials, there is a thorny photophysical problem called aggregation-caused quenching (ACQ): emission from a solution of luminescent molecule is partially or completely quenched once the luminophore aggregates (Mei et al., 2015). Obviously, the ACQ effect greatly hinders their applications of many optical materials. Taking optoelectronic materials as an example, in OLED real-world applications, luminophores are commonly used as thin solid films. Fortunately, in 2001, another photophysical effect called aggregation-induced emission (AIE) was reported by Luo et al. (2001). In the AIE process, non-emissive or faintly emissive luminophores are induced to display strong luminescence by the aggregate formation. Therefore, AIE-active luminogenic molecules have important applications in many fields. For example, Min et al. synthesized a benzothiadiazole-modified AIE-active water-soluble luminogen, which can combine with the pillar [5]arene by host-guest interaction. Notably, although this assembling system has a weak photodynamic activity in neutral microenvironment, this host-guest complex possesses outstanding targeted photodynamic therapy capacity in an acidic tumor microenvironment (Min et al., 2022). Zou et al. reported a highly-emissive AIE luminogen by integrating a luminogenic organic unit simultaneously exhibiting AIE and photosensitive features with an anticancer active gold(I) moiety. Excitingly, the introduction of gold(I) group not only enhances the photo-dynamic therapy efficiency of the organic ligand but also endows the good anticancer performance of this prepared mononuclear gold(I) complex (Zou et al., 2021).

**SCHEME 1:**
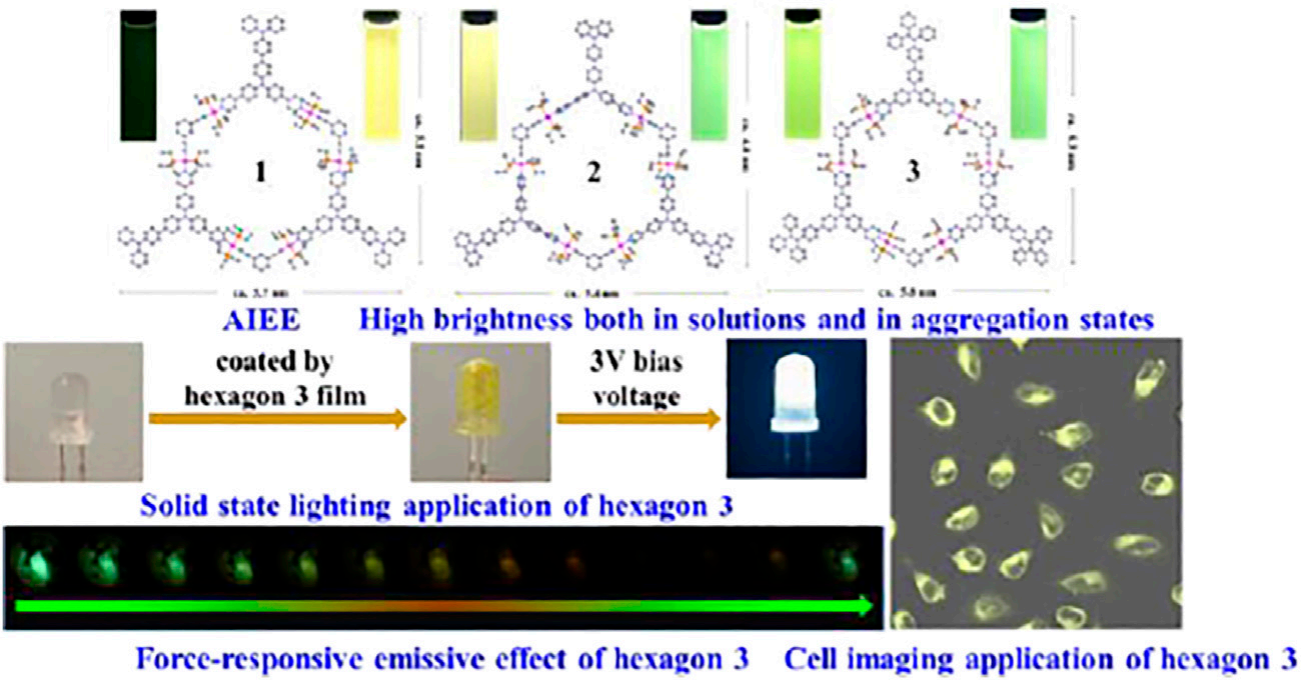
Schematic representation of highly emissive multifunctional materials (Yin et al., 2022).

Smart emitters that show external stimuli-responsive emissive properties have attracted increasing attention due to their significant applications in molecular switches, sensors, optical devices and anti-counterfeiting. Yin et al. reported a dianthracene-modified supramolecular organoplatinum (II) hexagon, and this fluorescent metallahexagon exhibited three-color mechanochromic and thin-film vapochromic fluorescence characteristics (Yin et al., 2021). Subsequently, Yin et al. developed a tetraphenylethylene-decorated highly emissive multipurpose platinum (II) metallacycle (Scheme 1), which could be applied as a coating for white lighting or as a stain for cell imaging. Additionally, this fluorescent metallacycle showed clear mechanofluorochromic response under mechanical stress (Yin et al., 2022). Wang et al. summarized the recent progresses of mechanoluminochromic metal-organic molecules including metal complexes and metallic clusters, and the corresponding mechanoresponsive mechanisms were also described. High brightness aggregative-state luminescence and high-color contrast before and after stimulation are considered as two vital indicators for achieving promising applications of stimuli-responsive luminogenic molecules. AIE luminogens with twisted molecular conformations and strong aggregative-state emission are important candidates of high-performance stimuli-responsive emissive materials. For example, Huang et al. prepared a tetraphenylethylene-functionalized AIE-active luminophor showing multi-state mechanochromic and photochromic behaviors, and this AIE luminogen could be used for multidimensional anti-counterfeiting (Huang et al., 2019). Tian et al. presented an aggregation-induced emission enhancement-active tetraphenylethene-based rhodanine derivative with good cell imaging performance, and this luminogenic compound possessed remarkable mechanochromic and solvatochromic fluorescence phenomena. Furthermore, the luminogen could be used for sensing Hg^2+^ on test paper strips.

In summary, this Research Topic has collected diverse aspects of stimuli-responsive emissive organic and metal-organic compounds. More specifically, the topic illustrates a variety of functionalized luminogenic molecules with different stimuli-responsive properties, and this relevant stimuli-responsive mechanisms are systematacially investigated and presented. Research on developing stimuli-responsive luminogenic materials is a fascinating subject, and the current Research Topic is anticipated to provide a valuable reference for the exploration of this hot research field.
